# Emerging Treatments in Bone Tumors: Lessons Learned from the ESMO Annual Meeting

**DOI:** 10.3390/biom16081167

**Published:** 2026-08-11

**Authors:** Samhita Kotapati, Meenakkshy Manoharan, Emanuela Palmerini

**Affiliations:** Sylvester Comprehensive Cancer Center, Miller School of Medicine, University of Miami, Miami, FL 33125, USA; sxk2220@miami.edu (S.K.); mxm195192@miami.edu (M.M.)

**Keywords:** bone sarcoma, osteosarcoma, antibody–drug conjugates, tumor microenvironment, tyrosine kinase inhibitors, Ewing sarcoma, immune checkpoint inhibitors, CAR T cell therapy, chondrosarcoma

## Abstract

Primary bone sarcomas are rare, heterogeneous malignancies with limited therapeutic options, particularly in metastatic disease where outcomes remain poor. This study synthesizes current literature to evaluate emerging therapeutic strategies and biological determinants of response across osteosarcoma, Ewing sarcoma, and chondrosarcoma, as presented at the ESMO Annual Meeting, 2025. Key approaches reviewed include VEGFR-targeted tyrosine kinase inhibitors (TKIs), DNA damage response (DDR) inhibition, MYC targeting, immune checkpoint inhibitors (ICIs), and surfaceome-directed therapies such as antibody–drug conjugates (ADCs) and chimeric antigen receptor T cell therapy. Across studies, TKIs demonstrated short lasting activity as monotherapy but improved outcomes in some of the studies when combined with ICIs or chemotherapy, reflecting their role in remodeling the tumor microenvironment (TME); importantly, controlled studies with TKI and chemotherapy upfront are ongoing. DDR- and MYC-targeted therapies have shown strong preclinical rationale but limited clinical efficacy, highlighting challenges in translation. Immune-based therapies exhibited variable responses, with dedifferentiated chondrosarcoma (DDCS) emerging as a responsive histotype. Surfaceome-targeting strategies, particularly ADCs, demonstrated promising early clinical activity. Overall, bone sarcoma rarity, tumor heterogeneity, immunosuppressive TME, and lack of predictive factors challenge drug discovery for bone sarcoma patients. These findings underscore the importance of combination strategies and biomarker-driven patient selection and suggest that continued integration of targeted and immunotherapeutic approaches will be critical to improving outcomes in bone sarcoma.

## 1. Introduction

Bone tumors are rare malignancies, with an estimated 4110 new cases expected to be diagnosed in the United States in 2026 [1]. Primary bone sarcomas account for approximately 0.2% of all malignant tumors and encompass a highly heterogeneous group of diseases, including numerous ultra-rare histologic subtypes such as leiomyosarcoma and BCOR-rearranged sarcomas, each with distinct biological and clinical characteristics [2]. Despite this diversity, osteosarcoma, Ewing sarcoma, and chondrosarcoma represent the most common entities, collectively accounting for approximately 90–95% of all primary bone sarcomas. Osteosarcoma is the most frequent, with an incidence of approximately three cases per million per year, followed by chondrosarcoma at two cases per million per year and Ewing sarcoma at one case per million per year. These subtypes also exhibit distinct age distributions, with median ages at diagnosis of 16 years for osteosarcoma, 50 years for chondrosarcoma, and 14 years for Ewing sarcoma [3].

Osteosarcoma and Ewing sarcoma, despite distinct genomic architectures, are both relatively chemo-sensitive tumors. Osteosarcoma is characterized by a highly complex karyotype with genomic instability, chromothripsis, and kataegis, whereas Ewing sarcoma exhibits a simple karyotype with marked epigenetic heterogeneity and a low somatic mutational burden. Clinically, both respond to multi-agent chemotherapy. In Ewing sarcoma, 5-year overall survival is approximately 84% with the induction chemotherapy with VDC/IE regimen (vincristine, doxorubicin, cyclophosphamide/ifosfamide, etoposide) delivered every 2 weeks, as compared with the VID chemotherapy every 3 weeks [4]. For osteosarcoma patients with localized disease, 5-year survival is more than 75.4% with Methotrexate, Doxorubicin, and Cisplatin (MAP) plus mifamurtide, or with mifamurtide combined with HDIFO in patients with a poor response to induction chemotherapy [5]. In contrast, chondrosarcoma is largely chemo resistant. Prognosis is primarily determined by tumor grade, with 5-year survival rates of approximately 93% for low-grade disease compared to 31% for high-grade tumors [6].

Contrary to localized disease, outcomes for patients with metastatic bone sarcomas remain poor, and there is no universally accepted standard of care. In osteosarcoma, 5-year survival in metastatic disease is approximately 29% [7]. In Ewing sarcoma, high-dose chemotherapy as consolidation has shown limited benefit; a 2022 study comparing standard therapy (VAC) with high-dose treosulfan and melphalan reported 5-year overall survival (OS) rates of 33.6% and 26.8%, respectively, demonstrating no clear improvement in patients with primary disseminated disease [8]. Chondrosarcoma, particularly in its dedifferentiated form, is associated with especially poor prognosis, with median OS as low as 6.8 months compared to 23.9 months in non-dedifferentiated disease [9].

Several prognostic factors have been identified in bone sarcomas, with the tumor microenvironment (TME) emerging as a key determinant of clinical outcomes. A recent large-scale study analyzing over 1300 sarcoma samples used integrated genomic and transcriptomic profiling and revealed substantial heterogeneity in immune infiltration across subtypes, confirming its association with survival. Immune-based clustering defined a continuum from “immune-cold” to “immune-hot” phenotypes, characterized by progressively increasing infiltration of T cells, B cells, dendritic cells, and myeloid populations. Notably, osteosarcoma, Ewing sarcoma, and chondrosarcoma predominantly exhibited immune “cold” profiles, which were associated with poorer OS [10]. Further supporting this, Palmerini et al. (2025) evaluated the prognostic significance of TME-related gene expression in osteosarcoma [11]. The authors identified a 21-gene TME signature that stratified patients into high- and low-risk groups with markedly different outcomes. The low-risk group demonstrated enrichment of cytotoxic and immune-active populations, including CD8^+^ T cells, regulatory T cells (Tregs), and activated natural killer cells, whereas high-risk tumors were characterized by increased CD4^+^ T cell-associated signatures. Correspondingly, 5-year OS was 92% in the low-risk group compared to 47% in the high-risk group [11]. These findings highlight the critical role of immune cell composition and trafficking within the TME as key determinants of prognosis in bone sarcomas and provide a strong rationale for therapeutic strategies aimed at enhancing immune infiltration. Against this backdrop, where first-line regimens, such as MAP and VDC/IE, have reached a therapeutic ceiling in the relapsed and metastatic setting, the emerging strategies reviewed here represent the next frontier of investigation; their incremental benefit must be interpreted in the context of these established benchmarks.

## 2. Materials and Methods

This narrative review is based on an Educational Lecture entitled Emerging Therapies in Bone Sarcoma delivered at the European Society for Medical Oncology (ESMO) Congress 2025. The content was developed from the original lecture materials, including slide decks and supporting references, which summarized recent advances in clinical trials, translational research, and precision-medicine approaches in primary bone sarcomas. Key studies discussed during the lecture were qualitatively reviewed and thematically synthesized according to disease subtype and therapeutic strategy, with emphasis on emerging targeted and immune-based treatments. This review reflects the authors’ interpretation of data presented at ESMO educational activities and does not represent an official ESMO position. As a narrative review derived from educational lecture, this work does not employ systematic search methodology, risk-of-bias assessment, or meta-analytic synthesis; evidence from Phase I, Phase II, Phase III clinical trials and preclinical studies is presented descriptively and thematically, and readers should interpret findings according to the corresponding level of evidence.

Additionally, generative artificial intelligence was used in this work. Specifically, Claude (Anthropic, San Francisco, CA, USA) was used to assist with figure preparation and diagram creation. Open Evidence (OpenEvidence Inc., Cambridge, MA, USA) was used to assist with grammatical editing, improvement of manuscript flow, and reference formatting. Reference management was initially conducted using Zotero (Corporation for Digital Scholarship, Vienna, VA, USA); citations were subsequently formatted and verified using a plain-text workflow. All AI-generated and AI-assisted content was critically reviewed and verified for accuracy and scientific integrity by the authors in accordance with the journal’s AI disclosure policy.

## 3. VEGFR-Targeted Multi-Kinase Tyrosine Kinase Inhibitors

**Figure 1:**
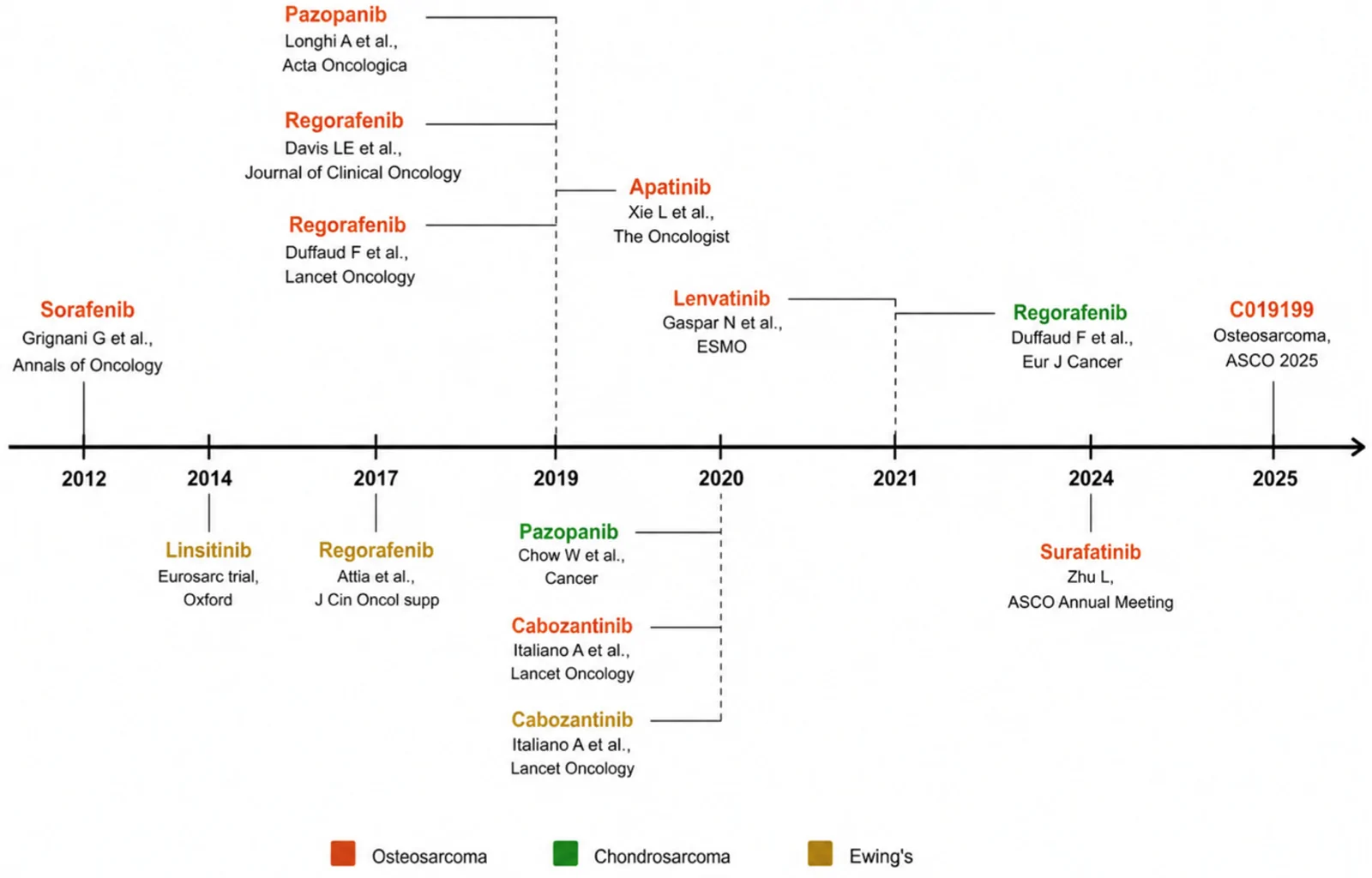
Timeline of pharmacological TKI development for osteosarcoma (orange), chondrosarcoma (green), and Ewing (yellow) [12,13,14,15,16,17,18,19,20,21,22,23].

VEGFR-associated multi-targeted tyrosine kinase inhibitors (TKIs) have been studied over the last decade as a therapeutic strategy for osteosarcoma, chondrosarcoma, Ewing sarcoma–including agents regorafenib, pazopanib, lenvatinib, and cabozantinib, as shown in Figure 1 [12,13,14,15,16,17,18,19,20,21,22,23]. These TKIs have produced modest benefits with objective response rates (ORR) generally below 15% and median progression-free survival (PFS) of 3–6 months [12,13,14,15,16,17,18,19,20,21,22,23]. Apatinib, a highly VEGFR2-selective agent, has shown higher overall response rates (43%) in Chinese cohorts without combination therapy [16]. More recently, emerging agents surufatinib and C019199 have incorporated CSF1-R inhibition to exploit the central role of CSF-1R in tumor-associated macrophages and M2 polarization to remodel the bone tumor’s immunosuppressive microenvironment to a more immune-permissive one [19,24,25,26].

In relapsed osteosarcoma, two VEGFR-selective TKIs have been evaluated in combination with ifosfamide and etoposide (IE). Lenvatinib plus IE achieved a 4-month PFS rate of 76.3% versus 66% for IE alone (hazard ratio 0.54; 95% CI 0.27–1.08; *p* = 0.04), though the wide confidence interval crossing 1.0 limits the statistical strength of the finding [27]. Apatinib plus IE demonstrated a more statistically convincing signal: 65.8% versus 41.6% PFS at 4 months (hazard ratio 0.60; 95% CI 0.37–0.98; *p* = 0.0402), with a confidence interval excluding 1.0 and qualifying it as a positive study [28]. The contrast between these two studies illustrates how similar absolute PFS differences can yield opposing statistical conclusions based on confidence intervals. Nonetheless, this data positions the apatinib + IE combination as the slightly stronger candidate for further investigation in relapsed osteosarcoma.

Beyond angiogenesis inhibition in TME modulation, cabozantinib has been shown to reduce Tregs, which normally suppress immune response and enhance CD8+ effector T cell activity and cytokine production [29,30]. Table 1 highlights its targeted receptors: MET, RET, KIT, and TAM (Tyro3, AXL, MER). In the CABONE phase II trial, cabozantinib resulted in a 12% ORR and a 33% 6-month non-progression rate (NPR) in osteosarcoma and a 26% ORR in Ewing sarcoma–the strongest single-agent response for a TKI in these histotypes at the time [18].

This success catalyzed further evaluation through clinical trials which will assess over 1700 patients with bone tumors, namely high-grade osteosarcoma, Ewing sarcoma and undifferentiated round cell sarcoma in the upfront setting. First, AOST2032 (NCT056-91478), a phase II/III trial coordinated by the Children’s Oncology Group, is evaluating cabozantinib in combination with MAP chemotherapy with follow-up maintenance in patients under 40 with newly diagnosed osteosarcoma [31]. Additionally, the European FOSTER Consortium (Fight Osteosarcoma Through European Research) is developing the FOSTER-CabOS trial, which will evaluate cabozantinib in newly diagnosed osteosarcoma patients following completion of MAP chemotherapy [32]. Lastly, regorafenib, a broad spectrum TKI inhibitor (Table 1), is being evaluated in combination with VDC/IE chemotherapy, showcasing a maximum tolerated dose (MTD) at 82 mg/m^2^/day, capping at 160 mg [33]. Building on this safety data, the Inter-Ewing-1 trial—will evaluate the efficacy of adding regorafenib to standard multimodal treatment in 900 patients with newly diagnosed metastatic Ewing sarcoma [33].

Also highlighted in Table 1, zanzalintinib represents a next-generation multi-targeted TKI targeting VEGFR2, MET, and the TAM kinases—an overlapping but more selective kinase profile than cabozantinib, with a shorter half-life that may improve tolerability. Preclinical and early clinical data suggest that the inhibition of MET and TAM kinases by zanzalintinib reprograms the TME to support immune activation and enhance responsiveness to immune checkpoint inhibitors (ICI), providing a mechanistic rationale for combination strategies [30,34]. And a phase II trial (ZAMBONE) will inform on the activity of zanzalintinib in advanced or metastatic patients with bone sarcoma [35].

Together with the cabozantinib trials in osteosarcoma (AOST2032 and FOSTER-CabOS) and development of zanzalintinib, Inter-Ewing-1 reflects a broader paradigm shift toward integrating TKIs into frontline therapy for primary bone tumors rather than reserving them for the relapsed setting.

## 4. DNA Damage Response Inhibition

WEE1 is a serine/threonine kinase that regulates the G2/M checkpoint by phosphorylating CDK1, preventing premature mitotic entry and maintaining genomic stability. WEE1 inhibition creates a state of “synthetic lethality” in TP53-mutant tumors, where inhibition forces TP53-mutant cells through mitosis with unrepaired DNA damage because they lack the p53-dependent G1 checkpoint [36]. A phase I trial examining the effects azenosertib, a highly selective WEE1 inhibitor, in combination with gemcitabine on recurrent or refractory (R/R) osteosarcoma found the 18-week event-free survival (EFS) to be 39% across all dose levels, exceeding its historical benchmarks for therapy in this setting [37]. The combination was well tolerated at the MTD (azenosertib 150 mg daily on a 5:2 schedule plus gemcitabine 800 mg/m^2^), supporting advancement to an investigator-initiated phase II trial [37].

PARP (poly-ADP ribose polymerase) repairs single-strand DNA breaks via the base excision repair (BER) pathway, and its inhibition exploits existing DNA repair defects in tumor cells via synthetic lethality, a mechanism shared with WEE1 inhibition as discussed above. In Ewing sarcoma, the EWSR1::FLI1 fusion protein confers heightened sensitivity to PARP inhibition in vitro and in vivo compared to other cancer cell lines, with Ewing cell lines demonstrating effective concentrations markedly lower than osteosarcoma or epithelial tumor lines [38]. The mechanistic basis for this sensitivity is multifactorial: EWSR1::FLI1 drives transcriptional R-loop accumulation that impairs BRCA1-mediated homologous recombination (HR), creating a BRCAness-like vulnerability. Additionally, EWS-FLI1 expression has been associated with suppression of Schlafen-11 (SLFN11), a replication stress response protein, and with synthetic lethality when combined with CDK12 inhibition—findings that have informed combinatorial preclinical strategies including PARPi with NAMPT inhibitors and CDK12/13 inhibitors [39,40].

Despite this compelling preclinical rationale, clinical translation has been poor. Combinations of trabectedin plus olaparib, and niraparib plus irinotecan or temozolomide, have failed to demonstrate significant benefit in Ewing sarcoma [41,42]. Several mechanisms likely underlie this translational gap. First, tumor cells may circumvent PARP inhibitor-induced synthetic lethality through upregulation of compensatory HR repair pathways–including RAD51-mediated strand invasion and alternative end-joining mechanisms–that restore genomic repair capacity independently of BRCA1 [43]. Second, PARP trapping, which underlies the cytotoxic mechanism of several PARPi agents, may be insufficient in the context of EWSR1::FLI1-driven chromatin remodeling, which alters the accessibility of PARP1-DNA complexes to trapping agents. Third, the absence of validated predictive biomarkers–such as homologous recombination deficiency (HRD) scores or SLFN11 expression–has precluded biomarker-driven patient selection, likely diluting observed clinical benefit across unselected populations [38,40,43].

The limited activity of PARPi combinations, specifically with Ataxia telangiectasia and Rad3-related protein (ATR) kinase inhibitor, in Ewing sarcoma appears to extend to osteosarcoma. ATR kinase is a central regulator of the DDR, sensing replication stress and activating cell-cycle checkpoints and repair pathways to preserve genomic integrity [44]. In a phase II trial of olaparib with ceralasertib (ATR kinase inhibitor) in R/R osteosarcoma, the 4-month EFS was approximately 15% with an ORR of 5.3%, which failed to meet the predetermined efficacy threshold [45]. In osteosarcoma, the heterogeneous and largely BRCA-proficient status of tumors further limits PARPi efficacy, as the absence of a homologous recombination deficiency phenotype removes the key molecular prerequisite for synthetic lethality. The lack of validated predictive biomarkers for DDR pathway defects in both histotypes has also hampered patient selection, potentially diluting the observed clinical benefit.

However, there are still active clinical trials in both Europe and the US in pursuit of a successful PARPi combination. In Europe, active arms within the AcSé-ESMART platform trial include Arm D (olaparib plus irinotecan) and Arm N (olaparib plus ceralasertib); a GSK-sponsored phase I study (SCOOP) is evaluating niraparib plus dostarlimab (anti-PD-1) in pediatric patients [46,47]. In the United States, Dana-Farber/Harvard Cancer Center is evaluating olaparib plus temozolomide (NCT01858168), Pediatric MATCH is assessing olaparib monotherapy, and St. Jude Children’s Research Hospital is investigating liposomal irinotecan combined with temozolomide or talazoparib [48,49,50]. Additionally, promising pre-clinical data shows a synergistic combination of PARPi and nicotinamide phosphoribosyl transferase (NAMPT) and between PARPi and CDK12/13 inhibitors in EWSR1::FLI1 lines [39,40].

Altogether, this data highlights the difficulty of translating successful DDR-targeted therapy preclinical data to successful clinical trial data for bone sarcomas, despite compelling preclinical data and principles of synthetic lethality. There is a current need for biomarker-driven patient selection and novel combinatorial approaches to benefit from WEE1, PARP, and ATR kinase inhibition strategies.

## 5. MYC Targeting

MYC amplification and overexpression are associated with poor prognosis in osteosarcoma. MYC copy number gains vary between 10–60% of patients depending on the methodology of study [51]; this data also includes a Children’s Oncology Group (COG) series of 137 patients of whom 12% expressed MYC amplification [52]. A methodological challenge across MYC studies is the absence of consensus of definitions for MYC genomic amplification and MYC protein expression. Amplification has been variably defined as a MYC-to-chromosome-8 copy number ratio > 2.0 [52] or an absolute copy number > 7 [53,54], while protein overexpression has been defined by H-score > 175 or assessed as a continuous variable [53,54]. Standardization of these definitions represents an essential prerequisite for cross-study comparability and the prospective validation of MYC as a stratifying biomarker.

Due to the poor prognosis association with MYC amplification/expression and osteosarcoma, researchers in Spain have developed and conducted phase I trials for the first-in-class direct MYC inhibitor OMO-103, a truncated form of the human MYC protein acting as a G-quadruplex stabilizer [55]. Currently, the OSTEOMYC phase II trial (NCT066-50514) sponsored by Vall d’Hebron Institute of Oncology is evaluating OMO-103 at the established recommended phase II dose (RP2D) of 6.5 mg/kg of OMO-103 as a weekly IV infusion in patients with relapsed osteosarcoma [56].

## 6. Immune Checkpoint Inhibitors

ICIs exploit tumor immune evasion, and their combination with TKIs has emerged as a rational therapeutic strategy in bone sarcomas given the established capacity of TKIs to remodel the immunosuppressive TME toward an immune-permissive state. In a phase II trial of nivolumab plus sunitinib in relapsed bone sarcomas, the 6-month PFS was 42%, mean PFS 3.8 months, and ORR 5% [57]. By histotype, dedifferentiated chondrosarcoma (DDCS) patients demonstrated a 25% ORR while osteosarcoma patients achieved a 6% ORR [57], underscoring DDCS as the most responsive histotype among bone sarcomas evaluated. In a dedicated DDCS cohort (*n* = 19), nivolumab plus sunitinib yielded 26.3% partial responses, 52.6% stable disease, and an intention-to-treat ORR of 21% [58].

Most strikingly, a Chinese cohort study of pembrolizumab plus anlotinib demonstrated an ORR of approximately 32%, among the highest reported for ICI + TKI combinations in bone sarcoma. The 6-month PFS was 57.1% versus 9.1% for anlotinib alone (*p* = 0.002) [59]. By histotype, DDCS showed the most substantial benefit—6-month PFS rising from 9.1% to 57.1% and 12-month OS from 18% to 42.9% [59]. Osteosarcoma and Ewing sarcoma remained largely refractory. The relationship between tumor mutational burden and immunotherapy response has been well established across solid tumors more broadly, where higher mutational burden is thought to generate more tumor neoantigens and improve responsiveness to immune checkpoint blockade [60]. Applying this framework to bone and cartilage sarcomas warrants caution, however, as the current evidence in DDCS and other bone sarcoma histotypes remains limited to single-arm or small-cohort trials rather than confirmatory phase III data, and TMB itself has not been systematically characterized across these histotypes. Collectively, while DDCS has a worse prognosis, it emerges as the most consistently responsive histotype across both ICI + TKI combination regimens evaluated.

## 7. Surfaceome Targeting

**Figure 2:**
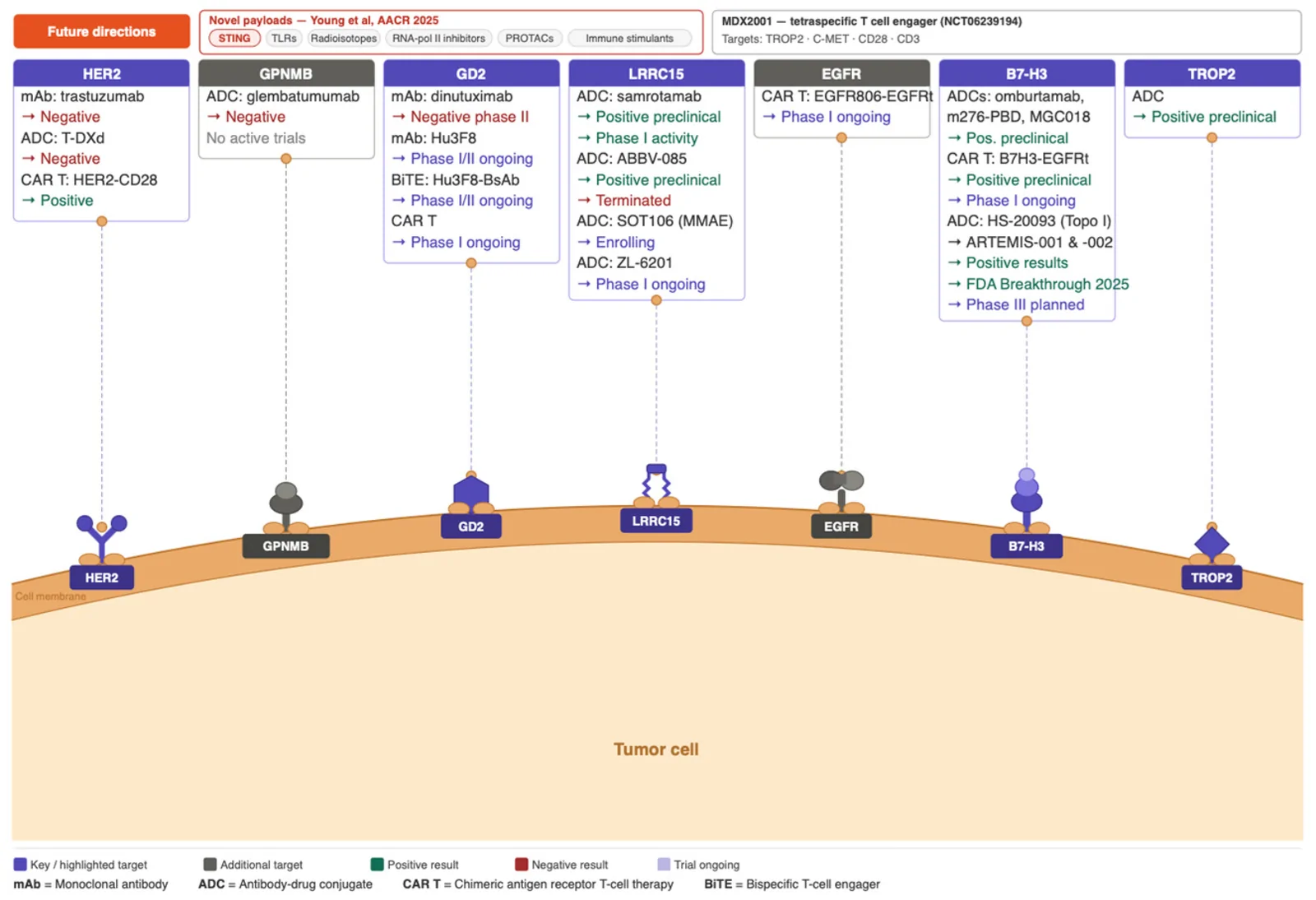
Emerging immunotherapeutic and targeted therapy strategies against bone sarcoma surfaceome—clinical and preclinical landscape [61,62,63,64,65,66,67,68,69,70,71,72,73,74,75,76,77,78,79,80].

Figure 2 highlights the major surfaceome receptors currently being targeted for therapeutic advantage and their trial status, positive results, and/or negative results.

### 7.1. Monoclonal Antibodies and Bispecific Strategies

Monoclonal antibodies (mAb) and bispecific strategies can leverage surfaceome targets to directly engage the immune system against tumor-associated antigens. Results for GD2-directed antibody strategies in osteosarcoma have been mixed. Naxitamab, a humanized IgG1 anti-GD2 mAb, yielded a 12-month EFS of 36% in pulmonary-only recurrent osteosarcoma; yet the wide 95% confidence interval (23–50%) reflects uncertainty [62]. Similarly, Nivatrotamab, a GD2 × CD3 bispecific antibody derived from the hu3F8 platform in patients with relapsed/refractory solid tumors, was evaluated in a phase I trial and demonstrated an acceptable safety profile; however, the study was terminated due to financial restructuring [63]. Lastly, a phase II trial for dinutuximab, a chimeric anti-GD2 mAb previously approved for neuroblastoma, failed to progress to Stage 2 as only 11 out of the 39 patients with recurrent osteosarcoma remained event-free at 12 months [64].

Omburtamab, a murine mAb targeting B7-H3 (CD276), represents a promising therapeutic candidate given that B7-H3 is expressed in 91.8% of osteosarcomas with limited expression on normal tissues [65]. Although a phase I trial specifically for osteosarcoma patients has yet to be conducted, a phase I study of intraperitoneal radio iodinated Omburtamab enrolled 52 patients with peritoneal tumors—including 48 with desmoplastic small round cell tumor, three with rhabdomyosarcoma, and one with Ewing sarcoma—and demonstrated a favorable safety profile with no dose-limiting toxicities across escalated activities ranging from 1.11 to 3.33 GBq/m^2^ [81].

Most recently, B7-H3 has been leveraged as a target for bispecific T cell engager (BiTE) strategies. Tamronlimab pelunstide (tam-peli), a B7-H3 × CD3 bispecific antibody, was evaluated in the ARTEMIS-II phase I/II study in patients with R/R EWS (*n* = 20) and chondrosarcoma (CHS; *n* = 10), all with metastatic disease and ≥1 prior line of systemic therapy [66]. Tam-peli demonstrated notable disease control in CHS—a histotype with very limited systemic options—achieving a disease control rate of 100% and a stable disease with any tumor reduction rate of 77.8% among nine evaluable patients, with one confirmed partial response (ORR 11.1%) and a median PFS of 11.7 months. At a median follow-up of 14.2 months, 70% of CHS patients remained alive, with median OS not yet estimable [66]. The safety profile was manageable, with Grade ≥ 3 TRAEs in 37.5% of patients overall and a treatment discontinuation rate of only 3.1%; no treatment-related deaths occurred [66]. These early data position B7-H3-directed bispecific T cell engagement as a complementary immunotherapeutic approach to B7-H3-directed ADCs, with clinical activity across both EWS and the historically chemorefractory CHS histotype.

### 7.2. Antibody–Drug Conjugates

In osteosarcoma, the overexpression of multiple cell-surface molecules has provided the biological rationale for antibody-based therapeutic approaches targeting the surfaceome—the complete collection of proteins expressed on cell membranes. Clinical evaluation of antibody–drug conjugates (ADC) in osteosarcoma has yielded heterogeneous results across targets [61]. For example, trastuzumab deruxtecan (T-DXd), a HER2-directed ADC with a topoisomerase I inhibitor payload, failed to meet the criteria for stage 2 in a phase II trial, with only one of nine patients achieving stable disease at 24 weeks [67].

In contrast, HS-20093—a B7-H3-directed ADC with a topoisomerase I inhibitor payload—has demonstrated compelling activity in osteosarcoma, culminating in FDA Breakthrough Therapy Designation in January 2025 based on the ARTEMIS-001 and ARTEMIS-002 trial results, with phase III evaluation currently planned. In phase II of ARTEMIS-002 (*n* = 23), HS-20093 achieved an ORR of 17.4% at 12 mg/kg with a PFS of 8.2 months at 8 mg/kg, with responses also observed in Ewing sarcoma and synovial sarcoma [68]. Updated data as of July 2025 (*n* = 45 evaluable osteosarcoma patients) demonstrate an ORR of 16.7% in adolescents and 20.8% in adults, a 6-month PFS of 65.9%, and a median duration of response of 19.8 months in adults and 5.65 months in adolescents [69]. The lower adolescent ORR may reflect a shorter follow-up duration rather than diminished activity, and continued accrual will be required to determine whether response rates converge with those observed in adults. GSK recently began a global phase I trial (NCT06551142) to validate these results beyond Chinese patient population [70].

LRRC15 represents a compelling therapeutic target in osteosarcoma, with expression reported in 77% of adult patients and 59% of pediatric patients exhibiting ≥10% LRRC15-positive cells [71]. Samrotamab vedotin is an ADC consisting of a mAb targeting LRRC15 conjugated to monomethyl auristatin E (MMAE), a potent tubulin inhibitor [72]. Importantly, MMAE’s cell-permeable “bystander effect” kills neighboring cancer cells even in the absence of LRRC15 expression, while simultaneously increasing immune infiltrate within the TME [72]. Of note, unlike HER2-directed ADCs, LRRC15-targeting ADCs act on both tumor cells and the immunosuppressive stroma, thereby directly addressing the TME barrier to effective therapy [73]. In early clinical evaluation, ABBV-085 (AbbVie) demonstrated manageable safety and a 20% ORR in sarcoma patients at 3.6 mg/kg every two weeks, comparing favorably to available chemotherapy ORRs of less than 10% [74]. While the AbbVie trial is no longer active, a similar agent, SOT106, targeting LRRC15 with MMAE, is currently in development specifically for soft tissue sarcoma and osteosarcoma, with the clinical trial actively enrolling patients with ≥10% LRRC15 tumor expression [71]. Further supporting target validity, the same study reported LRRC15 expression in 58% of chondrosarcoma patients [71]. Additional agents under investigation include ZL-6201, an LRRC15 ADC with a camptothecin payload designed to target the TME with precision, currently under evaluation in a Chinese cohort (NCT06776198) [75].

Among surfaceome proteins being explored as ADC targets, 5T4 has emerged as a particularly compelling candidate. As an oncofetal glycoprotein, 5T4’s silence in healthy adult tissue can be re-expressed in cancer, correlating with aggressive tumor behavior and epithelial-mesenchymal transition (EMT) [82,83]. Physiologically, 5T4 modulates Wnt signaling and regulates CXCR4 surface expression and CXCL12-mediated chemotaxis [82,83]. 5T4 has valuable therapeutic potential as it can not only deliver payloads to 5T4-expressing cells but also cause immune redirection by utilizing bispecific antibodies co-targeting 5T4 and co-stimulatory receptors to boost T cell-mediated killing [84]. This rationale inspired the production of TUB-030: a next-generation ADC targeting 5T4 with an exatecan (topoisomerase I) payload, developed based on Tubulis’ proprietary P5 conjugation technology, which has demonstrated effective and durable responses in a range of preclinical solid tumor models [85]. The 5-STAR 1-01 clinical trial will enroll up to 130 patients with advanced solid tumors in the US and Canada with the dose optimization cohort focusing on selected major cancer types including osteosarcoma and other 5T4-expressing tumors [85]. ACR246, a first-in-class next-generation 5T4 ADC, is also entering clinical evaluation for 5T4-positive solid tumors [86]. Overall, the field of 5T4 modulators holds great potential for future cancer treatment modalities, particularly in the context of combination therapies that could overcome resistance mechanisms and improve the therapeutic window.

Additionally, other notable ADC trials target Glycoprotein non-metastatic melanoma protein B (GPNMB) and trophoblast cell surface antigen-2 (TROP2). Glembatumumab vedotin, an ADC targeting GPNMB, was evaluated in a phase II trial (AOST1521) for patients with relapsed or refractory osteosarcoma. During Stage I, 19 out of 22 patients ended with progressive disease and one death from end organ failure occurred that was possibly related to Glembatumumab vedotin. Therefore, the results of Stage I failed to meet the threshold required to proceed to Stage II [87]. Separately, preclinical data suggest an ADC targeting TROP2 may have the potential to exert a bystander antitumor effect on parental osteosarcoma cells. Datopotamab deruxtecan is an ADC composed of a mAb targeting TROP2 conjugated to the topoisomerase I inhibitor Dxd. Preclinical studies demonstrated that datopotamab deruxtecan induced immunogenic cell death in vitro and demonstrated a bystander effect, reducing viability in neighboring TROP2-negative cells [88]. The mechanistic basis for this bystander activity may involve extracellular vesicle-mediated redistribution of cytotoxic payload from TROP2-positive to neighboring TROP2-negative cells. This extracellular vesicle-mediated framework has been described for ADC classes broadly and may also inform evidence grading when distinguishing preclinical bystander observations from confirmed clinical activity [76]. Therefore, this bystander effect has favorable implications for osteosarcoma tumors with heterogeneous TROP2 expression.

Future directions of ADCs include employing ADCs with novel payloads such as stimulator of interferon genes (STING). Targeted delivery of a STING payload with an ADC has been shown to produce tumor regression and decrease systemic immune activation, an advantage in an immunologically suppressive osteosarcoma TME [77]. Additionally, new strides are being made for LRRC15 radioimmunotheranostics, a strategy specifically targeting TGFβ-driven resistance in solid tumors like osteosarcoma. DUNP19, developed as a mAb, allows for molecularly precise radiotherapy to LRRC15-expressing cancer cells, affecting both direct tumor cell killing and TME [78].

### 7.3. CAR T Cell Therapy

Beyond mAb and bispecific strategies, chimeric antigen receptor (CAR) T cell therapy has emerged as a powerful cellular immunotherapy approach that leverages surfaceome target expression to direct sustained, personalized antitumor cytotoxicity in bone tumors, as shown in Table 2. Notably, HER2 has been recognized as a surfaceome target in osteosarcoma and Ewing sarcoma, providing the basis for a CAR T cell approach using an HER2/CD28 co-stimulatory construct. In a phase I study of autologous HER2 CAR T cells administered after lymphodepletion in patients with advanced sarcoma (NCT00902044), clinical benefit—defined as complete response or stable disease by RECIST criteria—was observed in 7 of 14 infused patients (50%). This included three complete responses (21%) and four patients with stable disease (29%), while seven patients had progressive disease (50%) [79]. Complete responses were observed predominantly in patients with lower disease burden prior to infusion, and TME analysis of responding tumors revealed enrichment of CD4, CD11c, and granzyme B expression, suggesting that baseline immune activation may correlate with durable response [89]. Despite this promising subset of responders, overall response rates remain modest, likely reflecting challenges including limited CAR T cell persistence, tumor antigen heterogeneity, and the heavily pre-treated nature of the study population.

Table 2 also summarizes active CAR T cell clinical trials in bone sarcoma, including those targeting emerging antigens such as EPHB4, FOLR1, and EGFR. Among these, EPHB4-targeted CAR T cell therapy uses a mutated ephrin B2 ectodomain—the natural EPHB4 ligand—as its targeting moiety, paired with a CD28 co-stimulatory signaling domain. It is manufactured via piggyBac transposon-based gene transfer, a non-viral approach that reduces manufacturing cost and complexity while achieving stable genomic CAR integration [89]. FOLR1 has been functionally implicated in osteosarcoma pathophysiology, and preclinical FOLR1-CART results show marked tumor suppression with FOLR1-CAR T vs. untreated controls over 200 days [90]. Fred Hutchinson Cancer Center is currently conducting an early-phase clinical trial of FOLR1-directed CAR T cell therapy (NCT07227571) [91].

EGFR is another compelling target, as it is expressed in approximately 82% of osteosarcomas and contributes to chemotherapy resistance by promoting starvation survival and counteracting the antiproliferative and pro-apoptotic effects of agents such as doxorubicin and methotrexate [99]. EGFR806-EGFRt CAR T cells utilize a binder based on the mAb806 epitope, which selectively recognizes tumor-specific untethered EGFR while sparing normal cells that express wild-type EGFR in its tethered conformation [92]. A phase I trial of EGFR806 CAR T cells in children and young adults with R/R solid tumors demonstrated an acceptable toxicity profile, with mixed responses observed in some patients [93].

### 7.4. Conventional Chondrosarcoma

Within the bone tumor therapeutic landscape, conventional chondrosarcoma poses a distinct therapeutic challenge as a chemotherapy-resistant histotype. IDH1 mutations and DR5 represent the two most actionable molecular targets identified to date. In a phase I study (NCT02073994), ivosidenib, a selective mutant IDH1 inhibitor, demonstrated a median PFS of 7.4 months and an ORR of 23.1% among 13 patients with advanced conventional chondrosarcoma, with notably durable responses (median duration of response 53.5 months) and manageable toxicity [100]. Olutasidenib, a second mutant IDH1 inhibitor, was subsequently evaluated in a phase Ib/II trial (NCT03684811) and conferred disease control in the conventional chondrosarcoma subgroup (*n* = 16), with a median PFS of 3.5 months, though no objective responses were observed [101]. The CHONQUER trial (NCT06127407) is a phase III, randomized, placebo-controlled study evaluating ivosidenib in grades 1, 2, and 3 IDH1-mutant conventional chondrosarcoma, with PFS in grade 1 and 2 patients as the primary endpoint [102].

In parallel, INBRX-109, a tetravalent DR5 agonist antibody targeting the extrinsic apoptosis pathway, demonstrated a median PFS of 7.6 months and an ORR of 6.5% in a phase I study—a modest but noteworthy signal in a chemo refractory disease [103]. The ChonDRAgon phase II randomized trial (NCT04950075), comparing INBRX-109 to placebo across all conventional chondrosarcoma grades (2:1 randomization), has completed enrollment, and results are anticipated [104]. Together, these parallel randomized trials mark a meaningful maturation of the therapeutic landscape for conventional chondrosarcoma and will clarify whether IDH1 inhibition or DR5 pathway activation represent viable treatment strategies for this historically treatment-resistant histotype.

## 8. Conclusions

Over the last 40 years, genomic instability, immunosuppressive TME, and marked tumor heterogeneity have made improving outcomes for bone sarcomas especially challenging. However, the therapeutic landscape is beginning to evolve with the development of surfaceome mapping, TKI-TME remodeling, and cellular immunotherapy. Among the most promising combination therapies pair TKIs with chemotherapy or ICIs or ADCs targeting sarcoma-associated surface proteins. Translating these molecular insights into meaningful clinical advances for osteosarcoma and other bone tumors will require not only continued pharmacological innovation but also expanded international collaboration, equitable access to clinical trials, and the acceleration of biomarker-driven late-phase studies.

## Figures and Tables

**Table 1:** TKI drugs, their targeted receptors, potency, and additional notes.

Drug	Main Targeted Receptors	Approx. IC50 (Potency)	Key Notes
Regorafenib	VEGFR1–3, PDGFR, FGFR, KIT, RET, RAF	VEGFR2 ≈ 4.2 nM	Broad Spectrum
Sorafenib	VEGFR2–3, PDGFRβ, RAF, KIT, FLT3	VEGFR2 ≈ 90 nM	Less potent on VEGFR
Cabozantinib	VEGFR2, MET, RET, KIT, TAM *	MET ≈ 1.3 nM VEGFR2 ≈ 0.035 nM	High potency on MET and AXL
Lenvatinib	VEGFR1–3, FGFR1–4, PDGFRα, RET, KIT	VEGFR2 ≈ 0.74 nM	Strong FGFR inhibition
Apatinib	VEGFR2 (selective), c-KIT, RET	VEGFR2 ≈ 1–10 nM	Highly selective for VEGFR2
Pazopanib	VEGFR1–3, PDGFRα/β, FGFR1/3, KIT	VEGFR2 ≈ 10 nM	Less potent than lenvatinib
Zanzalintinib	VEGFR2, MET, RET, KIT, TAM *	MET ≈ 0.5 nM (estimated)	In development; similar to cabozantinib but more selective
Surufatinib	VEGFR1–3, FGFR1, CSF-1R	VEGFR ≈ 1.7 nM FGFR1 ≈ 4.6 nM	Also inhibits CSF-1R
C019199	VEGDR2, Discoidin Domain Receptor (DDR), CSF-1R	VEGFR ≈ 14 nM DDR ≈ 40 nM CSF-1R ≈ 79 nM	Also inhibits DDR and CSF-1R

DDR1 = Discoidin Domain Receptor 1; * TAM receptor = Tyro3, AXL, and MER.

**Table 2:** Select CAR T cell clinical trials targeting bone and soft tissue sarcoma-related antigens [79,89,90,91,92,93,94,95,96,97,98].

Target	NCT	Trial Description
GD2	NCT03635632	C7R-GD2 CAR T—relapsed/refractory GD2+ cancers (GAIL-N)
GD2	NCT03373097	Anti-GD2—pediatric high-risk/relapsed neuroblastoma and GD2+ solid tumors
B7-H3 (3CAR)	NCT04897321	B7-H3-specific autologous CAR T—pediatric solid tumors
B7-H3	NCT06500819	Autologous B7-H3 CAR T—relapsed/refractory solid tumors
EGFR	NCT03618381	EGFR806 CAR T—recurrent/refractory solid tumors, children and young adults
HER2	NCT00902044	Anti-HER2-CD28 CAR T—advanced HER2+ sarcoma
EPHB4	NCT pending	EPHB4-CAR T (AP8901)—Ewing Sarcoma
FOLR1	NCT pending	FH-FOLR1—relapsed/refractory osteosarcoma (Fred Hutchinson)
IL12 (Attil12)	NCT05621668	First-in-human Phase I—T cell membrane-anchored IL12, bone and soft tissue sarcoma

## Data Availability

No new data were created or analyzed in this study. Data sharing is not applicable to this article.

## Abbreviations

The following abbreviations are used in this manuscript:

AbbVie	ABBV-085
ADC	Antibody–drug conjugate
ATR	Ataxia telangiectasia and Rad3-related protein kinase
CAR	Chimeric antigen receptor
COG	Children’s Oncology Group
DDCS	Dedifferentiated chondrosarcoma
DDR	DNA damage response
EFS	Event-free survival
EMT	Epithelial-mesenchymal transition
GPNMB	Glycoprotein non-metastatic melanoma protein B
HR	Homologous recombination
ICI	Immune checkpoint inhibitor
IE	Ifosfamide and etoposide
mAb	Monoclonal antibody
MAP	Methotrexate, Doxorubicin, and Cisplatin
MMAE	Monomethyl auristatin E
MTD	Maximum tolerated dose
NAMPT	Nicotinamide phosphoribosyl transferase
NPR	Non-progression rate
ORR	Objective response rate
OS	Overall survival
PARP	Poly-ADP ribose polymerase
PARPi	Poly-ADP ribose polymerase inhibitor
PFS	Progression-free survival
RP2D	Recommended phase 2 dose
R/R	Recurrent or refractory
STING	Stimulator of interferon genes
TAM	Tyro3, AXL, MER
T-DXd	Trastuzumab deruxtecan
TKIs	Tyrosine kinase inhibitors
TME	Tumor microenvironment
Treg	Regulatory T cell
TROP2	Trophoblast cell surface antigen-2
VDC/IE	Vincristine, doxorubicin, cyclophosphamide/ifosfamide, etoposide
